# Editorial: Reviews in psychiatry 2022: forensic psychiatry

**DOI:** 10.3389/fpsyt.2024.1383848

**Published:** 2024-03-01

**Authors:** Gary A. Chaimowitz

**Affiliations:** ^1^ Forensic Psychiatry Program, St. Joseph’s Healthcare, Hamilton, ON, Canada; ^2^ Department of Psychiatry and Behavioural Neurosciences, McMaster University, Hamilton, ON, Canada

**Keywords:** aggression, substance use disorder, terrorism, risk prediction, omega - 3 - fatty acid

I am pleased to introduce this edition’s Research Topic review in forensic psychiatry. The breadth and depth of the submissions in this review speak to the robust nature of research in forensic psychiatry. The subjects of the articles are wide ranging, speaking to the innovative questions being asked in the field.

Tomlin et al. tackle a controversial topic that is trending internationally. Forensic psychiatry has historically tried to find an ethical home between the competing principles of beneficence and autonomy, especially given the coercive underpinnings of most, even most enlightened, forensic psychiatry systems.

The rampant substance abuse epidemic and its impermeability to treatment as usual, continues to shock and dismay many in high middle- and low-income countries. It is not surprising then that there is an increased interest, including political interest, in mandatory substance use treatment for justice involved persons. This paper on mandatory substance use treatment in Germany provides some insight into the impact of this treatment on recidivism in this population. Key is their recommendation for further research with a focus on recovery and strength-based interventions. This paper provides guidance for much needed research-as our countries tackle substance use disorders. Mandatory substance use treatment clearly comes in many forms which provide opportunities to find a balance between choice and mandatory treatment, with recidivism only one of the outcomes we should measure.

Aggression sits as a key behaviour that is measured, predicted, managed and sometimes mitigated in forensic psychiatry practice. Being over-determined aggressive behaviours are difficult to understand and manage. Risk management strategies have utilized a variety of medications to treat the underlying illness or disorder contributing to aggression, or even simply targeting symptoms. Apart from treating the symptoms of schizophrenia and bipolar disorder, the use of medications to treat aggression generally has been variably successful and can be associated with significant troubling side effects. Chemical restraint can at times linger and evolve into “chemical restraining treatment”, raising significant ethical concerns. Choy looks at alternative strategies to reduce aggression, specifically the impact of the Omega 3 fatty acids, vitamin D, magnesium and zinc. Omega 3 levels have long been studied in many medical and nutritional domains, but the results give us a useful focus for future research. This review provides us with fresh non-pharmacological ideas as we continue to search for and understand manage aggression.

In in many countries art therapies have been phased out as treatment options in forensic psychiatry institutions or hospital, for a variety of reasons including financial and a paucity of research. The study by Abbing et al. addresses the limited research on the topic of art therapy, drama therapy and dance therapy. Their systemic review, thematic analysis and meta-analysis provides us with useful information. For example, the effect on risk and protective factors appears significant. They caution us as the analysis is explorative but again is a call then for more work on the effectiveness and mechanisms of change for arts therapies in forensic psychiatry. This is particularly welcome as sadly many programs in forensic psychiatry can take the form of a controlled hotel with medication administration as the main intervention.

Terrorism and violent extremism have been common topics of discussion, review, study and speculation as of late. This is an area which is complex and difficult to understand. Apart from broad conclusions, and sometimes politically slanted, we know very little about the psychopathology, if there is psychopathology at all, of perpetrators of terror or violent extremism. Most of the studies have only looked at men. Rakhshandehroo et al. in the systematic review tackle the topic of female perpetrators of terrorism and violent extremism. Terrorism and violent extremism are not purely a male domain and in fact women are in increasing percentage of perpetrators. Curiously many studies assume an element of psychopathology when somebody commits a terrorist or extremist motivated act. They are however silent on the purely politically motivated violent acts, and where these derive from. Clearly personality disorders feature in some studies but to the surprise of the authors there is little known about the psychopathology of the female terror and violent extremism perpetrators. Although prediction management and risk of terrorism and violence extremism is the business of many agencies, disciplines and professions, forensic psychiatrists are often called on to provide expert opinions. This controversial area begs for more research.

It is a pleasure to see the range of forensic psychiatry expertise as demonstrated by the articles in our review. Forensic psychiatry is a rapidly developing specialty and has moved beyond the medicolegal to other challenging areas. The authors in this review cover a wide range of topics and talk to the opportunities for more exploration and research as we try to improve our understanding of the many elements that make up the field of forensic psychiatry, particularly when it comes to trying to understand aggression and violence.

## Author contributions

GC: Writing – original draft, Writing – review & editing.

